# The Evolution and Application of Artificial Intelligence in Rhinology: A State of the Art Review

**DOI:** 10.1177/01945998221110076

**Published:** 2023-01-29

**Authors:** Ameen Amanian, Austin Heffernan, Masaru Ishii, Francis X. Creighton, Andrew Thamboo

**Affiliations:** 1 Division of Otolaryngology–Head and Neck Surgery, Department of Surgery, University of British Columbia, Vancouver, Canada; 2 Department of Otolaryngology–Head and Neck Surgery, School of Medicine, Johns Hopkins University, Baltimore, Maryland, USA

**Keywords:** artificial intelligence, rhinology, machine learning, computer vision, prediction, prognosis

## Abstract

**Objective.:**

To provide a comprehensive overview on the applications of artificial intelligence (AI) in rhinology, highlight its limitations, and propose strategies for its integration into surgical practice.

**Data Sources.:**

Medline, Embase, CENTRAL, Ei Compendex, IEEE, and Web of Science.

**Review Methods.:**

English studies from inception until January 2022 and those focusing on any application of AI in rhinology were included. Study selection was independently performed by 2 authors; discrepancies were resolved by the senior author. Studies were categorized by rhinology theme, and data collection comprised type of AI utilized, sample size, and outcomes, including accuracy and precision among others.

**Conclusions.:**

An overall 5435 articles were identified. Following abstract and title screening, 130 articles underwent full-text review, and 59 articles were selected for analysis. Eleven studies were from the gray literature. Articles were stratified into image processing, segmentation, and diagnostics (n = 27); rhinosinusitis classification (n = 14); treatment and disease outcome prediction (n = 8); optimizing surgical navigation and phase assessment (n = 3); robotic surgery (n = 2); olfactory dysfunction (n = 2); and diagnosis of allergic rhinitis (n = 3). Most AI studies were published from 2016 onward (n = 45).

**Implications for Practice.:**

This state of the art review aimed to highlight the increasing applications of AI in rhinology. Next steps will entail multidisciplinary collaboration to ensure data integrity, ongoing validation of AI algorithms, and integration into clinical practice. Future research should be tailored at the interplay of AI with robotics and surgical education.

Artificial intelligence (AI) has been quickly expanding within the health care domain, as it utilizes complex algorithms and sophisticated computation to perform human cognitive tasks at astronomical speed.^1–3^ Given its promise for transforming medicine, it has seen increasing applications ranging from disease diagnosis to prognostication, treatment planning, and optimization of surgical efficiency.^3–5^ Due to its rapid and ongoing development, it is vital for a clinician to be aware of recent advances and consider its application in surgical practice.

Machine learning (ML) and deep learning (DL) are subsets of AI, which have recently become more commonplace due to the increased availability of computational power (Supplemental Table S1, available online).^1^ Additionally, the presence of big data has given ML the capability to make clinical predictions by identifying patterns within data, typically not identifiable by humans.^1^ Furthermore, such algorithms utilize techniques that identify nonlinear relationships among data variables and in various settings, and they have demonstrated superior performance when compared with traditional statistics.^1^ DL employs computations in multiple layers with methodologies that perform automated image segmentation or delineate phases within a surgical operation.^6^ Within otolaryngology, DL’s applications have ranged from image segmentation for diagnosis of maxillary sinusitis to differentiation of inverted papilloma (IP) from IP with malignant transformation.^7,8^

Due to the popularity of AI and its novel research outcomes, previous publications have introduced AI and its applications within otolaryngology.^9–11^ Rhinology is a subspecialty that has seen a myriad of technological advances, such as image-guided surgical navigation.^12^ Therefore, it is no surprise that there has been an increasing number of AI research within rhinology, given its promise to augment surgical practice and enhance patient-centered care.^13^

Although integration of technological advances such as ML, DL, and computer vision into current rhinologic practice is vital, it is still in its infancy, and the otolaryngologist must understand its fundamentals and potential surgical application. This state of the art review aims to review the current literature related to applications of AI in rhinology, discuss existing limitations, highlight areas of promise, identify gaps for future research, and provide otolaryngologists with an overview of the applications of AI in rhinology.

## Methods

A preliminary search on AI in rhinology was done, which yielded a small number of articles. Therefore, a scoping review was conducted to capture a wider breadth of articles. The review protocol was published on the Open Science Framework (doi:10.17605/OSF.IO/5K2GB). The reporting of this scoping review was conducted in accordance with the PRISMA-ScR statement (Preferred Reporting Items for Systematic Reviews and Meta-analyses Extension for Scoping Reviews).^14^ Risk of bias was not assessed in this review; however, the quality of literature was assessed via levels of evidence defined by the Oxford Centre for Evidence-Based Medicine.^15^

### Data Sources and Search Strategy

To identify relevant articles, a search was performed with the following databases from the date of inception until January 2022: MEDLINE, Embase, Cochrane Central Register of Controlled Trials, Ei Compendex, IEEE, and Web of Science Core Collection. The ClinicalTrials.gov registry was also screened for related ongoing trials. Gray literature was included, specifically conference proceedings identified from database and registry searches. The search strategy was developed with the assistance of 2 institutional medical librarians and consisted of 2 concepts: AI (ML, DL, neural network, computer vision, and robotics) and rhinology. These concepts and their related terms and synonyms were combined through relevant Boolean operators (Supplemental Appendix A, available online). The search strategy was modified for syntax where required.

### Study Selection

All publications obtained from the databases were exported to a systematic review management software (Covidence; Veritas Health Innovation Ltd). Duplicate studies were removed by this software. Articles were included if they focused on AI applications in rhinology. Exclusion criteria were as follows: AI in histopathology, nonautonomous robotic surgery, non-English studies, secondary literature, in vitro or animal studies, and lack of abstract or full-text access.

Search results were reviewed independently by title and abstract by 2 authors (A.A. and A.H.) with resolution of discrepancies by the senior author (A.T.). Short-listed articles were reviewed in full for eligibility by the 2 reviewers (A.A. and A.H.).

A final list of articles from each reviewer was compared and combined. A final list was presented to the supervising author for approval. Authors were not blinded to the journal, authors, or institution.

### Main Outcomes and Data Extraction

The main outcomes extracted revolved around AI performance and included area under the curve, sensitivity, specificity, accuracy, Dice similarity coefficient, and *F* score. The Dice similarity coefficient is an accuracy metric used in medical image segmentation that measures degree of overlap between 2 volumes and ranges from 0 (no overlap) to 1 (perfect overlap).^16^ *F* score, a measure of accuracy, attempts to combine the precision and recall within a model.^17^ Overall, the measured outcomes allowed authors to comment on the accuracy of AI technologies in rhinology. Reviewers extracted relevant data from the articles in duplicate to reduce bias and error. Data were extracted with a predefined template that included study type, level of evidence, author, year, objective, AI type, sample size, methodology, and performance outcomes. No uncertainties arose during the data extraction process.

### Synthesis of Results

Given the heterogeneity present in the studies within this review, a meta-analysis was not performed. Furthermore, the data were synthesized in a narrative fashion.

## Results

### Characteristics of Included Studies

**Figure 1.**
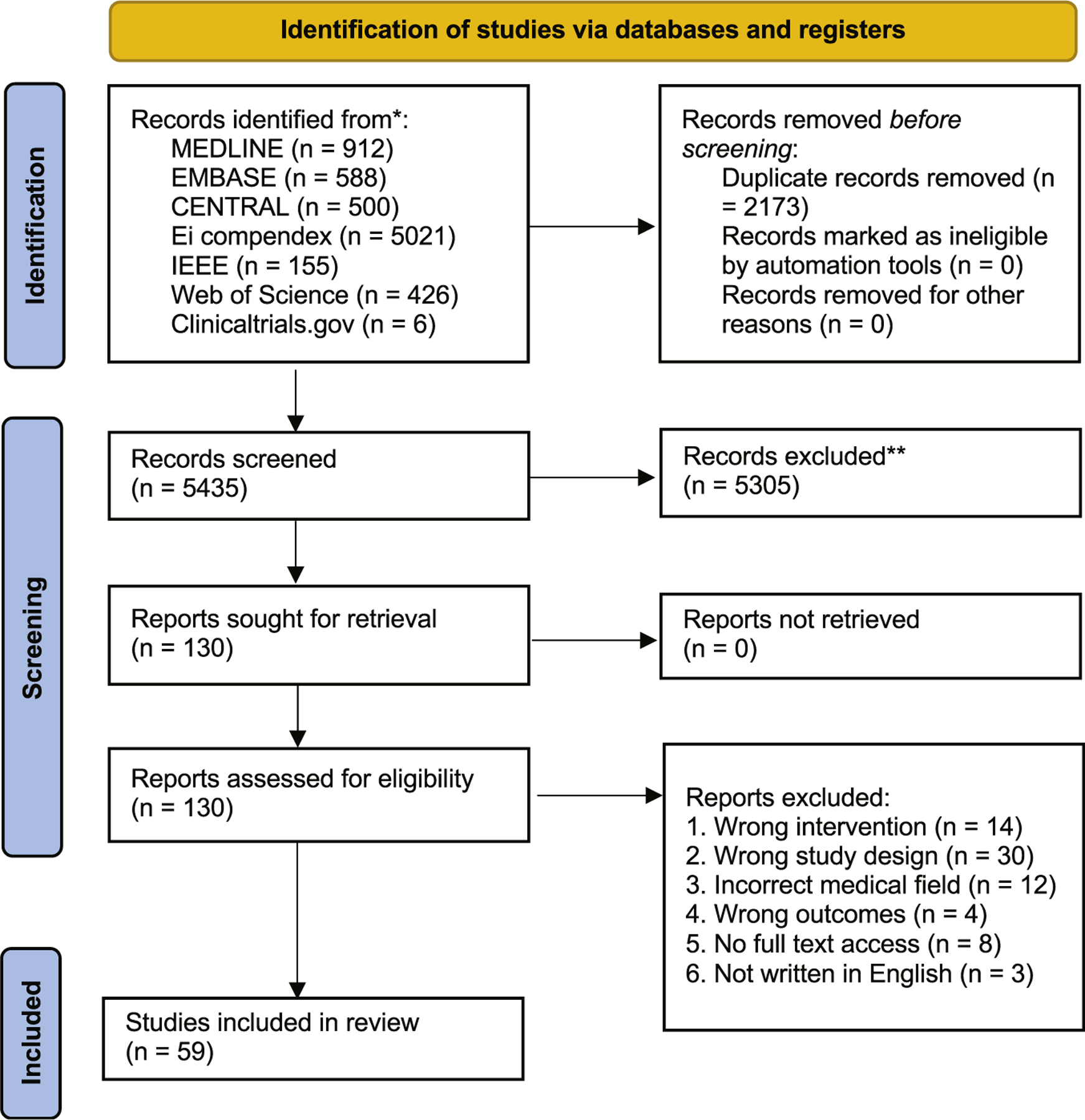
PRISMA flowchart of study identification.

**Figure 2.**
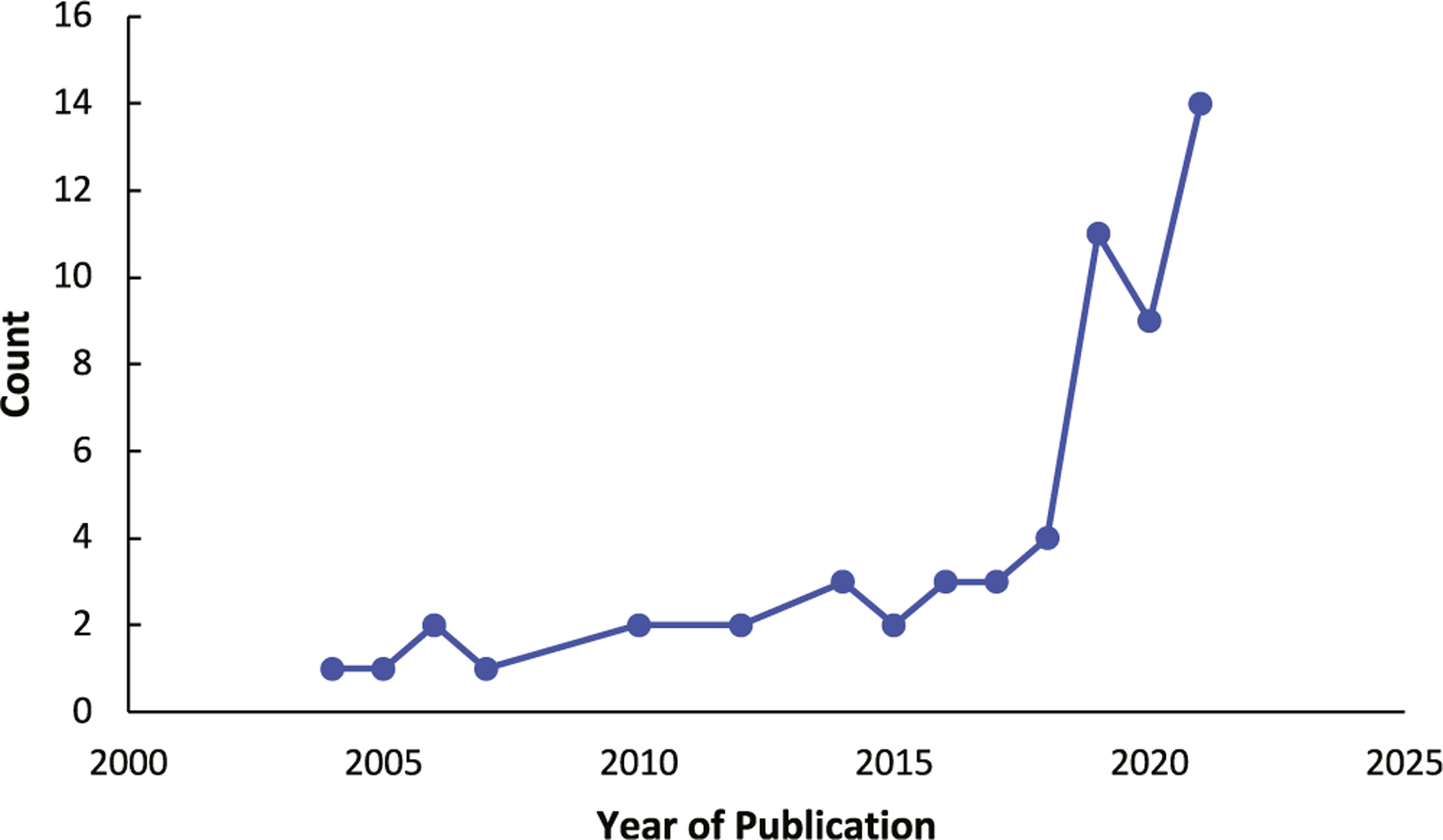
Trend of publications focusing on the application of artificial intelligence in rhinology.

The search strategy yielded 5435 articles after duplications were removed. A total of 5305 articles were excluded during title and abstract screening due to the publications not meeting the inclusion criteria or successfully fulfilling the exclusion criteria. This resulted in 130 articles that were sought for retrieval and assessment for eligibility (Figure 1). Articles were excluded due to wrong intervention (n = 14), study design (n = 30), outcomes (n = 4), incorrect medical field (n = 12), lack of full-text access (n = 8), and non-English language (n = 3). After exclusion of these articles, 59 were included in this review, with 11 coming from gray literature, mainly conference proceedings. These were stratified into various categories (Supplemental Tables S2-S9, available online): image processing, segmentation, and diagnostics (n = 27); rhinosinusitis classification (n = 14); treatment and disease outcome prediction (n = 8); optimizing surgical navigation and phase assessment (n = 3); robotic surgery (n = 2); olfactory dysfunction (n = 2); and diagnosis of allergic rhinitis (n = 3). The frequency of publications increased with time, as 76% of publications occurred from 2016 to 2021, 16% from 2010 to 2015, and 8% from 2004 to 2009 (Figure 2). This reflects the increased relevance and popularity of AI applications within rhinology in recent years.

### Level of Evidence

The level of evidence based on the Oxford Centre for Evidence-Based Medicine pertaining to the included studies can be referenced in the tables for each category.^15^ As expected, there was significant heterogeneity in the study design, objective, and results. Overall, the level of evidence ranged from 2 to 5.

## Discussion

### Review of AI

In general, AI is broadly separated into ML and natural language processing.^9^ The subsets of AI are briefly introduced to serve as a primer prior to summarizing the results of this review (Supplemental Table S1, available online). However, more in-depth information on the components of AI can be found in a review article written by Bur et al.^9^ Within ML, subsets can be divided into supervised learning and unsupervised learning.^3^ ML models are typically developed by splitting a data set into a training and testing set.^18^ In supervised learning, each point within a data set has an associated label, and the model is then validated via assessment of the testing set.^18^ Within otolaryngology, supervised learning has been used for classification of disease, including diagnosis of peritonsillar abscess,^19^ prediction of hearing outcome following sudden sensorineural hearing loss,^20^ and detection of oropharyngeal carcinoma,^21^ among others. Unsupervised learning, however, aims to identify patterns from unlabeled data.^22^ For example, clustering, a form of unsupervised learning, assesses an unlabeled data set to identify clusters to which a patient population may belong.^22^ This can especially be useful when there are subtle differences present within the study population typically difficult to directly discern.^22^ Finally, DL has recently seen an uprise in use due to the advent of computational power and availability of large data sets.^23^ DL algorithms can be used for classifying an image for diagnostics (eg, tumor vs no tumor) or segmentation (delineating a region of interest within an image such as a tumor).^23^ In otolaryngology, DL has shown an ability to predict extranodal extension with high accuracy^24^ and detect thyroid nodules on ultrasound images.^25^ The advantage of DL is the ability to automate image segmentation and classification and avoid the manual labor of image labeling; however, training of such algorithms requires a large data set, manual work up-front, and advanced computational throughput.^2^

Several themes were identified in this state of the art review. The studies have therefore been synthesized into categories in which otolaryngologists come across in their daily clinical practice (Supplemental Tables S2-S9, available online).

### Image Processing, Segmentation, and Diagnostics

Our review identified 27 studies that were in the realm of image segmentation and classification (Supplemental Tables S2-S4, available online). Within the realm of computer vision, DL has demonstrated an ability to differentiate neoplasms such as IP from IP with malignant transformation,^7^ segment nasopharyngeal carcinoma,^26^ and classify IP vs nasal polyps on preoperative computed tomography (CT) scans.^27^ Li et al developed a nasopharyngeal malignancy detection model from endoscopic images using a fully convolutional network.^28^ The detection model was able to perform tumor detection in a much shorter time as compared with manual segmentation and outperform experts.^28^ Therefore, in surgical oncology, DL can provide clinicians with further information in the preoperative setting for diagnosis^29,30^ and the postoperative setting for monitoring of recurrence.^31^ With ongoing advancements in AI, it remains to be seen whether the pre- and postoperative oncologic care that patients undergo will evolve with time.

Prior to sinonasal surgery, the team performs a thorough evaluation of patient imaging to identify anatomic variations, assess extent of sinonasal disease, and devise a surgical plan for addressing the sinonasal disease.^32^ With the goal of reducing intraoperative complications, DL can serve as a tool in classification of anatomic variation, disease identification, and surgical planning.^23^ For example, convolutional neural networks have been used in detecting osteomeatal complex occlusion for 2-dimensional coronal CT images,^33^ predicting the location of the anterior ethmoid artery as within the mesentery or skull base,^34^ and identifying a concha bullosa at the level of the osteomeatal complex.^35^ However, such cohorts have included only 2-dimensional images, which makes the transferability difficult given that 3-dimensional scans are used within the clinical setting.^33^ Nevertheless, DL tools demonstrate immense potential for enhancing preoperative evaluation and thereby reducing the risk of surgical complications.

DL algorithms have been used to diagnose sinusitis^36–38^ or quantify sinus volumes^39–42^ on radiographic imaging. In fact, algorithms have shown superior accuracy in the diagnosis of maxillary sinusitis when compared with the performance of radiologists^41^ or dental residents.^43^ In addition, studies have extended their scope to diagnose sinusitis in other sinuses (eg, frontal or ethmoid) with acceptable results.^44,45^ As obtaining a large data set is not always feasible, one group was able to demonstrate high performance when diagnosing maxillary sinusitis within a smaller data set using a transfer learning approach.^46^ Additionally, DL algorithms can be used for grading disease severity^47^ to determine surgical candidacy or to detect anatomic structures within the nasal cavity^17,48–50^ for surgical planning and medical education. Overall, this shows the promise of AI solutions in providing diagnostic and teaching support to clinicians and trainees.

### Classification of Rhinosinusitis

The continued advancement in science has prioritized personalized medicine, which entails clustering patients into certain groups to streamline and determine the optimal treatment modality.^51^ This is certainly evident in the evolution of chronic rhinosinusitis (CRS) diagnosis.^52^ Recently, CRS subsets have migrated from the traditional CRS with and without polyps to the modern clustering of cases according to anatomic location and endotypes.^52^ The observational nature that leads to disease clustering is the hallmark of unsupervised learning: a process that aims to identify patterns through observation of data as opposed to being provided a label for each data point.^53^ Its utility has been demonstrated for cases such as predictions of patient phenotype or health status.^3,22,54^

This review highlights several studies of unsupervised learning to determine patient clusters within CRS (Supplemental Table S5, available online).^55–59^ Parsel et al used 22 variables, such as demographics, quality of life domains (eg, SNOT-22), comorbidity scores, and disease diagnosis, to place patients in 7 distinct clusters.^55^ Although most diagnoses were correlated with 1 cluster, some (eg, CRS without nasal polyposis) was associated with multiple patient clusters possibly due to differences in disease endotypes.^55^ Divekar et al studied the use of the preoperative SNOT-22 survey for clustering CRS cases.^56^ Interestingly, the last 2 clusters were associated with a lack of aspirin hypersensitivity, while the last cluster had minimal symptomatic improvement following surgery.^56^

Unsupervised learning has shown to successfully cluster patients with allergic rhinitis,^60^ CRS with and without nasal polyposis,^58,61^ and olfactory dysfunction.^62^ Other forms of ML have been implemented to predict eosinophilic CRS^57^ or distinguish controls from patients with bacterial sinusitis by using a collection of exhaled gas from the nasal airway.^63^ Random forest models found IL-5 and IL-13 cytokines to be most predictive of olfactory dysfunction in patients with CRS who were undergoing surgery.^62^ Nevertheless, studies that have aimed to classify and differentiate among forms of sinusitis^64–69^ may have immense potential to improve surgical care.

### Treatment and Disease Outcome Prediction

With the advent of electronic medical records and curation of medical databases, supervised ML algorithms have been increasingly applied within health care settings for providing disease predictions.^70^ In the setting of CRS, factors such as preoperative SNOT-22 and disease phenotype have been used for predicting postoperative outcomes^71,72^ (Supplemental Table S6, available online). Chowdhury et al used a random forest algorithm demonstrating preoperative SNOT-22 and several cytokines, including IL-5 and TNF-a, to be important predictors of postoperative SNOT-22 scores.^73^ As our understanding of CRS has evolved, its multifactorial nature has become evident.^52^ Therefore, ML has a role to play in this realm given its ability to parse complex interrelationships among clinical variables.^74^

Using known clinical variables to provide predictions is an avenue to be explored with ML. ML algorithms have been trained and shown potential in distinguishing treatment responders vs nonresponders in acute rhinosinusitis,^75^ differentiating postoperative quality of life following identification of patient clusters,^76^ and predicting treatment outcomes in different patient cohorts.^77–79^ Kim et al found that with Lund-Mackay score and age, the number of subepithelial human neutrophil elastase was predictive of surgical outcomes in patients who had CRS with nasal polyps.^80^ ML models can also identify the importance of each variable within the prediction model and enhance its clinical interpretability.^81^

With health care’s mission toward personalized medicine, ML algorithms identifying patients at risk of treatment failure can be used to strive for a preventative care model.^82^ Fujima et al studied the use of quantitative magnetic resonance imaging variables in identifying local control vs failure in patients diagnosed with sinonasal squamous cell carcinoma and achieved high sensitivity and specificity.^83^ Moreover, a neural network was superior in predicting risk of nasopharyngeal carcinoma recurrence as compared with traditional statistical methods such as logistic regression.^84^ As ML predictive tools integrate into clinical practice, they can be used for disease prevention and prediction of recurrence.

### Optimizing Surgical Navigation and Surgical Phase Assessment

Image-guided surgery was a major milestone in the evolution of rhinology. It allowed for improved dissection and surgical navigation while reducing risk of injury to nearby critical structures.^85^ There has been ongoing investment of research in achieving submillimeter accuracy given the current registration accuracy of 2 mm.^86^ As a result, computer vision methods have been explored to improve traditional registration methods.^87,88^ Three studies were highlighted in this subcategory (Supplemental Table S7, available online). Reiter et al developed a learning-based video CT registration algorithm providing 3-dimensional reconstructions of the sinonasal cavity during endoscopy.^87^ This work was improved with implementation of a self-supervised convolutional neural network method to register intraoperative videos with CT scans achieving submillimeter accuracy.^88^ Finally, surgical phase assessment has been recently explored to help with predicting surgical steps, avoiding complications, and providing feedback to surgeons.^6^ Bieck et al implemented a natural language processing technique predicting future surgical steps from the current endoscope location.^89^ Further studies in this realm may focus on providing surgeons with the optimal surgical pathway and even predicting the ideal instrumentation depending on the anatomic region that is being operated on.

### Robotic Surgery

Robotic surgery has made significant advances in other domains of otolaryngology, such as management of oropharyngeal tumors, while attaining reduction in complications and postoperative morbidity.^90^ Due to the bony barriers and small nasal aperture, its current application in rhinology has been limited.^90^ We included studies within robotics if they had a component of full or semiautomation relevant to the task (Supplemental Table S8, available online). Steinhart et al constructed a robot that was able to successfully follow a path and perform automated resection of the anterior wall of the sphenoid sinus.^91^ To provide surgeons the ability to operate with 2 hands, Dai et al designed an automated endoscope holder utilizing a tracking algorithm.^92^ With the development of flexible and miniature instruments, the use of complex ML algorithms, and the integration of image-guidance systems, the next evolution in rhinology may be with the advent of robotic sinonasal surgery with various automation features.^90^

### Olfactory Dysfunction

ML algorithms were utilized among several studies to determine associations between sinonasal inflammation and olfactory dysfunction^62^ (Supplemental Table S9, available online). Morse et al studied a CRS group, identifying 5 patient subgroups in which they characterized inflammatory patterns and studied their association with olfactory dysfunction.^62^ Within the patient clusters, there were statistically significant differences in Smell Identification Test scores.^62^ Thereafter, applied ML models found IL-5 and IL-13 cytokines to be most predictive of olfactory dysfunction in patients with CRS who were undergoing surgery.^62^ Similarly, unsupervised analysis has been studied to classify specific clusters within viral rhinitis cases based on olfactory function scoring systems.^93^ Clustering techniques are beneficial as specific patient clusters can be followed in a prospective setting to study whether other factors may contribute to olfactory dysfunction. This is especially relevant with the COVID-19 pandemic, as smell and taste loss has served as a predictor of COVID-19 infections.^94^ As long-term data continue to be curated, ML may have a role in identifying patients at risk of long-term olfactory dysfunction,^95^ establishing routine follow-up, and offering intervention should olfactory dysfunction develop.

### Diagnosis of Allergic Rhinitis

Although allergic rhinitis is one of the most common allergic presentations, patients require an individualized treatment approach.^96^ However, given the various allergens and other potential etiologies, the timely diagnosis and treatment of this common condition may be a challenge.^96^ Therefore, clinical decision support tools aimed to aid clinicians with this diagnosis may be of benefit. For example, Jabez Christopher et al compared various supervised learning approaches and developed a tool for the diagnosis of allergic rhinitis via results of intradermal skin tests.^97^ Interestingly, the tool had a diagnostic accuracy of 88% vs 58.2% when compared with junior clinicians.^97^

Given the increasing incidence of allergic rhinitis, especially in children, AI can identify risk factors to help with disease prevention.^98^ Using a random forest approach, Huang et al found prenatal air quality to be an important predictor of developing childhood allergic rhinitis.^98^ Unsupervised techniques can also be used to identify patients who may develop severe forms of allergic rhinitis.^60^ Therefore, AI can identify risk factors for developing allergic rhinitis, and with public health measures, we can move toward a preventive medical approach for rhinologic conditions (Supplemental Table S9, available online).

## Conclusions and Implications for Practice

AI is quickly gaining traction and becoming a popular area of research within otolaryngology.^9,99^ Studies to date have mainly focused on rhinosinusitis classification and image processing and segmentation. This has been partly due to the enhanced computational power and ability to train and test on complex data sets.^1,2,23^ Future areas of research will certainly be extended into robotic surgery within rhinology, especially with the goal of reducing surgical morbidity, lowering a surgeon’s cognitive load, and enhancing surgical dissection and patient outcomes. Additionally, DL has a role in identifying phases within a surgical procedure, providing performance metrics, and serving as a tool for trainee education.^6,89^

AI studies within rhinology have shown promise to augment clinical practice by aiding clinical diagnosis and allowing clinicians to focus on delivering empathetic care to patients. Supervised learning may be incorporated into the clinical setting to help clinicians infer a provisional diagnosis.^55,100^ As we move toward personalized medicine, clustering patients into disease endotypes or classifying disease from large data sets or various imaging modalities in an efficient manner is of utmost importance. However, as consideration is given to the integration of AI algorithms into the clinical domain, we must confirm data integrity, use large data sets to ensure generalizability, and validate the algorithm using external data sets.^101^ Therefore, multi-institutional collaborations are needed given the requirements of big data while preserving patient confidentiality.^99^ Importantly, it must be iterated to clinicians that AI will assist them and augment their practice instead of replacing human intelligence.^102^

Most publications to date have been within the research setting, and the question remains how to incorporate ML technologies into clinical practice.^103^ This is especially challenging with the ‘‘black box’’ component of ML^101^ and the lack of regulatory frameworks for evaluating AI algorithms.^103^ Furthermore, ensuring that clinical judgment is not biased from an algorithm’s suggestion is an ongoing area of discussion within the AI community.^99,101^ Finally, it is vital to confirm lack of bias within the original data set for training algorithms to ensure that the results are robust and generalizable. For example, dermoscopic images with the presence of a ruler were more likely to be deemed malignant as at baseline, malignant images more frequently had a ruler in the image.^104^ Nevertheless, ML can be a tool to complement health care practice and support clinical intuition.^1^

There are several limitations to the current study. First, histopathology studies were excluded given that otolaryngologists rarely interpret pathology and cytology slides. Second, studies that did not focus on the applications of AI in rhinology were excluded, which may have removed studies that had a minor AI component. However, our literature encompassed multiple clinical and engineering databases to be as comprehensive as possible for this review. Additionally, the references of systematic reviews and relevant studies were cross-checked for any other studies that may have fit the inclusion criteria. Finally, we included gray literature publications to ensure that studies in their early research phases were still afforded consideration in this review given the infancy of the applications of AI in rhinology.

We conducted a comprehensive state of the art review on the application of AI in rhinology. To date, this field has evolved with the introduction of image-guided surgery and advanced instrumentation.^85,86,90^ There is no doubt that it will continue to evolve with the continued evolution and translation of AI. As additional AI-centered research is conducted, AI interest groups should be established at institutional levels to ensure data integrity, patient data confidentiality, and continued validation of algorithms.^101^ Ongoing education in this area will ensure that otolaryngologists can parse through the technicalities of AI research and products, understand the clinical application of a proposed algorithm, and work toward integrating it into their clinical practice as a clinical decision support tool.

## Supplementary Material

Supplemental information
