# Supplementary material for: The Evolution and Application of Artificial Intelligence in Rhinology: A State of the Art Review
Source: Otolaryngol Head Neck Surg. Author manuscript; Available in PMC 2024 May 22. (PMC11110957; doi:10.1177/01945998221110076)
Supplement: Supplemental information [file NIHMS1986163-supplement-Supplemental_information.docx]

**Table S1:** Definitions of relevant terms used in artificial intelligence

| **Term** | **Definition** |
| --- | --- |
| 1. Artificial Intelligence | Use of computer algorithms and advanced modeling to automate cognitive processes^1,2^. |
| 1. Machine Learning | Utilizes a diverse set of algorithms to learn from previous data to make predictions on new data^1,3^. |
| 1. Supervised learning | Every point within a dataset has an associated label with a target value that the algorithm is trained on to then validate via a testing set^4^. |
| 1. Unsupervised learning | Identifies patterns and clusters from unlabeled data^5^. |
| 1. Neural Network | Subset of supervised learning where the output of one layer becomes the input for the next layer until the data is distilled to a perceptible output^3,6^. |
| 1. Clustering Analysis | Form of unsupervised learning that aims to identify clusters in which a patient population belongs to^1^. |
| 1. Deep Learning | Subset of ML where an algorithm with multiple layers (>3) is used to classify data such as images^6,7^. |
| 1. Artificially Intelligent Robotics | Robots that use ML algorithms to independently complete a task using data collected from their environment^6,8^. |
| 1. Natural Language Processing | Language and text are processed into information that can be comprehended by computer algorithms^1^. |
| 1. Random Forest Models | Algorithm that creates a series of decision trees from included variables to predict an outcome^9^. |
| 1. Support Vector Machine | Machine learning algorithm for pattern recognition in complex data sets^10^. |
| 1. Computer Vision | Artificial intelligence algorithms used for image and pattern recognition^3^. |

**Table S2:** Image Segmentation

| **Author, year** | **Application** | **SS** | **AI Type** | **Outcomes** | **LOE** |
| --- | --- | --- | --- | --- | --- |
| 1. Huang 2019 | Nasal cavity segmentation framework to automatically segment and produce nasal models for CFD | 30 | AIRS using Elastix | Framework achieved segmentation accuracy of 0.909 DSC | 4 |
| 1. Tang 2021 | Image segmentation platform for nasopharyngeal carcinoma segmentation from MRI images | 95 | Dual attention based DSNIS | Platform achieved segmentation accuracy of 0.805 DSC | 4 |
| 1. Neelapu 2017 | Automatic segmentation approach for segmentation of pharyngeal and sino-nasal airways on 3D CBCT data | 15 | Automated image segmentation algorithm | Achieved F-score > 80% (accuracy measure) using all segmentation techniques | 4 |
| 1. Jung 2021 | Segment maxillary sinus disease using 3D CBCT: segmenting maxillary sinus into bone, air, and lesion | 123 | CNN (3 steps: better performance each step) | Algorithm has a segmentation accuracy of 0.93 DSC for air and 0.76 DSC for lesion | 4 |
| 1. Humphries 2020 | DL for automated segmentation of paranasal sinuses to facilitate volumetric quantification of sinus cavity | 690 | CNN | CNN automated the sinus segmentation and is correlated with Lund Mackay (rho = 0.82) | 4 |
| 1. Sensakovic 2010† | Method for automated segmentation of mucosal change | 4 | Automated volumetric segmentation | Method achieved a segmentation accuracy of 0.61 DSC | 4 |
| 1. Passera 2006† | Semi-automatic segmentation algorithm for volumetric analysis of paranasal sinus and nasal cavity cancers | 64 | Semi-supervised Fuzzy C-means clustering | Detected no ES involvement or volume errors and corresponded with GT at accuracies of 84.4%, 89.1% and 79.7% | 4 |

* AI: Artificial intelligence; AIRS: Automated Image Registration-Segmentation; CBCT: Cone-beam computed tomography; CFD: computed fluid dynamics; CNN: Convoluted neural network; DL: Deep learning; DSC: Dice Similarity Coefficient; DSNIS: Dense SU-net image segmentation; ES: essential structures; GT: Ground truth; LOE: Level of evidence; MRI: Magnetic resonance imaging; SS: Sample Size; SFCM: Semi-supervised fuzzy-C-means; †: Grey literature

**Table S3:** Image Anatomical Structure Identification

| **Author, year** | **Application** | **SS** | **AI Type** | **Outcomes** | **LOE** |
| --- | --- | --- | --- | --- | --- |
| 1. Kuo 2020 | Automatic recognition and volume calculation for the inferior turbinate and maxillary sinus by using image processing techniques | 79 | BPNN | BPNN attained an IT and MS detection accuracy of 96.3% and Sn 95.1% | 4 |
| 1. Laura 2019† | DL to detect variable structures (nasal cavity and paranasal sinuses) | 57 | Darknet-19, YOLO | DL algorithm detects structures with increased accuracy if they are not divided into left and right | 4 |
| 1. Chowdhury 2019 | Robust CNN used to classify osteomeatal complex occlusion on CT | 239 | CNN | Osteomeatal complex occlusion detection accuracy 85% and AUC 0.87 | 4 |
| 1. Huang 2019 | CNN used to differentiate location of the AEA as either adhered to the skull base or within a bone ‘mesentery’ on coronal sinus CT scans | 972 | CNN | CNN was 82.7% (CI 77.7-87.8) accurate with AUC 0.86 at detecting AEA location | 4 |
| 1. Giacomini 2019 | Automated tool for quantifying the total and air free volume of the maxillary sinus based on CT images | 30 | Linear regression and image processing | Mean % difference of AvM for total MS and air-free MS was 7.19% ± 5.83% and 6.93% ± 4.29% respectively | 4 |
| 1. Parmar 2020 | CNN algorithm to determine MT pneumatization (concha bullosa) on coronal sinus CT images | 447 | CNN | CNN algorithm detects MT pneumatization with accuracy of 81.0% (73.0-89.0) and AUC 0.93 | 4 |
| 1. Natsheh 2010† | Neural networks-based software system for the analysis and diagnosis of sinus conditions. | 30 | Neural network | Sinus diagnostic system achieved an ROI maximum error of 8.8% | 4 |
| 1. Tingelhoff 2007† | Manual or semi-automatic segmentation of paranasal sinuses and nasal cavity for ENT surgical workflow | 98 | 3D growing region | Semi-automatic segmentation reduced ST by 745 min compared to manual segmentation | 4 |

* AEA: anterior ethmoid artery; AI: Artificial intelligence; AUC: Area under the curve, AvM: Automated vs. manual; AVS: Automated volumetric segmentation; BPNN: Back propagation neural network; CNN: Convolutional neural network; CT: Computed tomography; DL: Deep learning, DSC: Dice Similarity Coefficient, IT: Inferior turbinate; LOE: Level of evidence; MS: Maxillary sinus; MT: middle turbinate; ROI: Region of interest, Sn: Sensitivity, Sp: Specificity, SS: Sample size, ST: Segmentation time; †: Grey literature

**Table S4:** Image Disease Diagnostics

| **Author, year** | **Application** | **SS** | **AI Type** | **Outcomes** | **LOE** |
| --- | --- | --- | --- | --- | --- |
| 1. Kuwana 2021 | DL object detection technique in the detection of maxillary sinuses, and in the classification of maxillary sinus lesions and healthy maxillary sinuses on panoramic radiographs | 1174 | Neural network | DL detected MS with Acc 90-91%, Sn 88-85% and Sp 91-96%, DL detected MS cysts with Acc 97-100%, Sn 80-100% and Sp 100% | 4 |
| 1. Oh 2021 | End to end process in medical imaging that uses an independent task learning algorithm to evaluate its performance in maxillary sinusitis applications | 2122 | DL: YOLOv2 | Algorithm detected MS with Acc (AUC) of IS 88.93% (0.89) and ExS 85.13-91.67% (0.85-90). It diagnosed sinusitis with Acc (AUC) for IS 79.87% (0.80) and ExS 73.85-84.67% (0.74-0.82) | 4 |
| 1. Ren 2021 | DL framework for automatic recognition of IP and NP by CT images | 136 | CNN | DL framework was 89.3% accurate (AUC 0.95) at classifying IP and NP | 3 |
| 1. Liu 2022 | Using a 3D CNN to preoperatively distinguish benign inverted papilloma tumors from IP-SCC | 90 | 3D CNN | 3D CNN distinguished IP and IP-SCC with Sn 66.7%, Sp 81.5%, Acc 77.9% and AUC 0.8 | 4 |
| 1. Kim 2019 | Diagnostic performance of a DL algorithm in diagnosing MSi on Waters’ view radiographs | 9,000 | DL Algorithm | DL diagnosed MSi with an AUC TET set and GET set of 0.93 and 0.88 | 4 |
| 1. Murata 2019 | Deep-learning system for diagnosis of maxillary sinusitis on panoramic radiography | 12,000 | DL Algorithm | DL system diagnosed MSi with Acc 87%, Sn: 86.7%, Sp 88.3 % and AUC: 0.875 | 4 |
| 1. Ogawa 2021 | Texture analysis used to diagnose and differentiate ONB and SCC on contrast enhanced CT images | 43 | UMA (ElasticNet) | MAEN Acc overall was 86%, and 96% and 71% to detect SCC and ONB | 4 |
| 1. Mori 2021 | Detection of maxillary sinuses and diagnosis of maxillary sinusitis on panoramic radiographs | 610 | DL Algorithm | DL T25 model detected maxillary sinus with performance of 0.987 and T50 model diagnosed MSi with Acc 0.925, Sn 0.9 and Sp: 0.95 | 4 |
| 1. Jeon 2021 | Diagnosis of frontal, ethmoid, and maxillary sinusitis on both Waters’ and Caldwell views | 1532 | DL Algorithm | Algorithm detected FS, ES and MSi with AUC (95% CI) of 0.71 (0.62-0.80), 0.78 (0.72-0.85) and 0.88 (0.84-0.92) respectively | 4 |
| 1. Ramkumar 2017 | Utilizing MRI–based texture analysis to classify cases of non-coexistent SCC and IP | 46 | MRI–based texture analysis | MRI–based texture analysis classified SCC and IP with VT accuracy of 89.1% | 4 |
| 1. Chang 2005 | 3D Hopfield neural network called the spatiotemporal Hopfield neural cube for recurrent NP detection | 26 | Neural network | Neural network detected NP with Sn: 99.98% and Sp: 99.61% | 4 |
| 1. Li 2018 | AI tool to detect nasopharyngeal malignancies under endoscopic examination based on DL | 1430 | Fully CN | CN detected malignancy with Accuracy 88.0, Sp 85.5%, Sn 90.2% and AUC 0.938 | 4 |

* Acc: Accuracy; A AI: Artificial intelligence; UC: Area under the curve; CNN: Convolutional neural network; CN: Convolutional network; DL: Deep learning; ES: ethmoid sinusitis; ExS: External set; FS: frontal sinusitis; GET: Geographic external test; IP-SCC: inverted papilloma malignant transformation to squamous cell carcinoma; IP: inverted papilloma; IS: Internal set; LOE: Level of evidence; MS: Maxillary sinus; MSi: maxillary sinusitis; MAEN: Multivariate Analysis-ElasticNet; NP: Nasal papilloma; ONB: olfactory neuroblastoma; P: performance; SS: Sample Size; SCC: squamous cell carcinoma; TET: Temporal external test, UMA Univariate and multivariate analysis VT: Validation and training

**Table S5:** Rhinosinusitis Classification

| **Author, year** | **Application** | **SS** | **AI Type** | **Outcomes** | **LOE** |
| --- | --- | --- | --- | --- | --- |
| 1. Kim 2019 | Unsupervised cluster analysis of CRS with nasal polyposis using preoperative and postoperative clinical parameters | 375 | UC | UC yielded 2 asthmatic clusters and 4 non-asthmatic clusters | 4 |
| 1. Soloviev 2020 | Tool that allows for distinguishing between normal and two forms of chronic rhinitis: atrophic and hypertrophic | 78 | KNN, random forest, GBT, SVC and logistic regression | GBT achieved binary Acc of 98% and diagnostic Acc of 94% | 3 |
| 1. Thaler 2006 | Using an electric nose to distinguish between patients with and without bacterial rhinosinusitis | 79 | SVM (ML) | SVM (ML) detected bacterial rhinosinusitis with Acc 72% to 98.4% | 4 |
| 1. Rustam 2021† | Kernel entropy based fuzzy c-means clustering techniques to diagnose a patient with acute or chronic sinusitis | 6 | KEFCM | KEFCM diagnosed acute or chronic sinusitis with Acc 97% | 4 |
| 1. Divekar 2014 | UNA to characterize local and systemic immune responses associated with acute sinonasal symptoms in CRSwNP | 19 | UNA: force direct algorithm + weighted degree centrality | No accuracy data | 4 |
| 1. Nuseir 2021† | To classify sinus disease using a sinonasal diseases dataset, feature extraction, selection methods and ML | 50 | SVM, NB, DT (J48), KNN1, KNN5 | Classified sinus disease using SVM, NB, J48, KNNI and KNN5 with Acc 66%, 68%, 66%, 48% and 64% respectively. | 4 |
| 1. Arfiani 2019† | Utilizing AI to diagnose acute sinusitis | 200 | KSPKM, SVM | KSPKM and SVM diagnosed sinusitis with ACC of 97% and 90% | 4 |
| 1. Rustam 2019† | Propose kernel perceptron for sinusitis classification by modifying perceptron algorithm using kernel function. | 200 | Kernel perceptron | Classified sinusitis using 7-fold CV with Acc 96.94%, Sn 96.94% and Sp 96.94%, and 10-fold CV with Acc 97.39%, Sn 97.78% and Sp 97.0% | 4 |
| 1. Thorwarth 2020 | Utilizing machine learning to predict eosinophilic CRS. | 80 | ANN and LR | Predicting eCRS using LR (SD) and ANN (SD) achieved AUC of 0.945 and 0.956 | 4 |
| 1. Soler 2014 | Identifying phenotypic subgroups from a prospective cohort of patients with CRS and classifying patients into clusters. | 382 | UC | UC identifies CRS phenotypic subgroups with accuracy of 89% | 4 |
| 1. Lal 2018 | To identify features predicating outcomes from endoscopic sinus surgery in patients with CRS without nasal polyposis. | 146 | Unsupervised network modeling | 4 unique clusters were identified using this unsupervised network modeling | 4 |
| 1. Divekar 2015 | Using SNOT22 on preoperative data to understand CRS symptom heterogeneity and characterize clinical features. | 97 | Symptom-Based Clustering | 5 clusters were identified, and two clusters were associated with ASA sensitivity. | 4 |
| 1. Yeh 2012† | Electrodermal screening device used to diagnose allergic rhinitis. | 178 | KNN algorithm and SVM classification | AR detected by KNN and SVM with Acc of 93.26% and 97.78%. AR severity detected by SVM with Acc of 99.57% | 2 |
| 1. Parsel 2021 | AI used to differentiate patterns in patient data to develop clinically meaningful diagnostic groups. | 545 | UNHCA (PAM method) | UNHCA detected 7 unique patient clusters | 4 |

* Acc: Accuracy; AHC: Agglomerative hierarchical clustering; AI: Artificial intelligence; ANN: Artificial neural network; ASA: Aspirin; CRS: Chronic Rhinosinusitis; CV: Cross Validation; DT: Decision tree; GBT: gradient boosting decision trees; KMC: K-means clustering; KNN: K-Nearest neighbour; KSPKM: Kernel spherical K-Means; KEFCM: Kernel Entropy Based Fuzzy C-Means; LOE: Level of evidence; LR: Logistic regression ML: Machine learning; NB: Naïve bays; PAM: partitioning around medoids; SD: Surgeon selected datasets; SS: Sample Size; SVM: Support vectors machine; SVC: support vector clustering; UNA: Unsupervised network analysis; UC: Unsupervised clustering, UNHCA: Unsupervised non-hierarchical cluster analysis; †: Grey literature

**Table S6:** Treatment and Disease Outcome Prediction

| **Author, year** | **Application** | **SS** | **AI Type** | **Outcomes** | **LOE** |
| --- | --- | --- | --- | --- | --- |
| 1. Kim 2021 | Investigate surgical outcome predictors for CRSwNP patients with focus on neutrophilic localization | 129 | Decision trees and random forest | Mean accuracy (random forest) for correct prediction of good outcomes after surgery in CRSwNP was 84 % | 4 |
| 1. Qi 2021 | Modeling approach to understand heterogeneity of treatment effects by identifying subgroups with a deviated response | 24,392 | Synthetic random forest models | 4 subgroups were identified using 20 pre-treatment variables | 4 |
| 1. Chowdhury 2020 | Evaluating the role of mucus cytokines in predicting SNOT22 scores after ESS in a prospective cohort of CRS patients | 147 | Random Forest Algorithm | Prediction accuracy of post-op SNOT22 scores was variable dependent (Mean squared error percent (MSE%)): Preop SNOT: 41.5%, IL-5: 20.9%, TNF-alpha: 14.6%, IL-2: 10.4%, IL-13: 10.2%, Polyp: -4.74%, IL-21: -7.15%, IL-1B: -8.57% | 3 |
| 1. Fujima 2019 | Predicting treatment outcomes from ML algorithm combining MRI-derived data in sinonasal SCC patients | 36 | Non-linear SVM | Local control predicted with accuracy 92%, Sn 100%, Sp 82%, PPV 0.82 and NPV 1.0 | 4 |
| 1. Szaleniec 2014 | Multidimensional models to predict early outcomes of ESS in individual patients | 115 | Network analysis | Early outcomes of ESS detected with Sn 93% and Sp 86% | 4 |
| 1. Szaleniec 2016 | Prediction of the intensity of facial pain following surgery for CRS | 139 | Artificial Neural Networks | Intensity of postoperative facial pain detected with Sn 93% and Sp 86% | 4 |
| 1. Kumdee 2012 | A generalized Neural Network-type SIRMs method to predict nasopharyngeal carcinoma recurrence | 495 | GNNtSIR Modules connected fuzzy inference method | Nasopharyngeal carcinoma recurrence detected with AUC 0.8 and accuracy 80% | 4 |
| 1. Adnane 2017 | To identify phenotype and mucosal eosinophilia endotype subgroups of patients with medical refractory CRS and evaluate difference in quality-of-life outcomes after ESS between these clusters | 131 | Unsupervised Two-step Cluster Analysis and logistic regression model | Three clusters identified and logistic regression model explained 55.6% of change in postoperative quality of life | 3 |

* AI: Artificial intelligence; AUC: Area under the curve; CRS: Chronic Rhinosinusitis; CRSwNP: Chronic Rhinosinusitis with Nasal Polyps; ESS: Endoscopic sinus surgery; GNNtSIR: Generalized Neural Network-type Single Input Rule; IL: Interleukin; LOE: Level of evidence; ML: Machine learning; NPV: Negative predictive value; PPV: Positive predictive value; R2: R-Squared; SS: Sample Size; Sn: Sensitivity; Sp: Specificity; SVM: Support vector machine; TNF: Tumour necrosis factor

**Table S7:** Optimizing Surgical Navigation and Phase Assessment

| **Author, year** | **Application** | **SS** | **AI Type** | **Outcomes** | **LOE** |
| --- | --- | --- | --- | --- | --- |
| 1. Liu 2020 | Self-supervised approach to train CNN for dense depth estimation of monocular endoscopy data without a priori modeling of anatomy or shading | 10 | CNN: All-Net | CNN estimated depth with Sn 66.7%, Sp 81.5%, AUC 0.80 and overall accuracy of 77.9% | 4 |
| 1. Reiter 2016 | Method for dense reconstruction of anatomical structures using white light endoscopic imagery based on a learning process that estimates a mapping between light reflectance and surface geometry | 206 | Novel learning process that estimates mapping between light reflectance and surface geometry | Reconstructs structures with mean (SD) accuracy of 0.53-1.12 mm (0.38-0.69) | 4 |
| 1. Bieck 2020 | Predicting endoscope positions along the navigation process in surgery and to estimate future endoscope positions | 3850 | NS: Transformer model  SG: ABSA | NS accuracy of transformer model was 53% and SG accuracy for ABSA was 83% | 4 |

*ABSA: Adapted beam search algorithm; AI: Artificial intelligence; CNN: Convolutional neural network; LOE: Level of Evidence; NS: Navigation steps; SD: Standard deviation; SG: Sentence generation; Sn: Sensitivity; Sp: Specificity; SS: Sample Size

**Table S8:** Robotic Surgery

| **Author, year** | **Application** | **SS** | **AI Type** | **Outcomes** | **LOE** |
| --- | --- | --- | --- | --- | --- |
| 1. Steinhart 2004 | A novel robot system designed with remote control systems and surgical instruments, adapted especially to meet the demands of paranasal sinus surgery | 5 | Integrated robotic system, A73 (Computer navigation-guided, fully automated, and telemanipulation) | Successful performance of automated sphenoidectomy on cadaveric heads | 3 |
| 1. Dai 2016† | A robot system that can move in the nasal cavity automatically with little surgeon interference | 0 | 7 degrees of freedom robotic endoscope holder | Robot system achieved a max (x, y) tracking error of 1.93 deg and max (z) tracking error of 2.43 deg. | 5 |

* AI: Artificial intelligence; LOE: Level of evidence; SS: Sample Size; RMS: root mean square; †: Grey literature

**Table S9:** Olfactory Dysfunction **and Diagnosis of Allergic Rhinitis**

| **Author, year** | **Application** | **SS** | **AI Type** | **Outcomes** | **LOE** |
| --- | --- | --- | --- | --- | --- |
| 1. Morse 2019 | Validate patterns of olfactory dysfunction in CRS using hierarchical cluster analysis, machine learning algorithms and multivariate regression. | 110 | Hierarchical cluster analysis and random forest approach | IL-5 and IL-13 most predictive of OD | 4 |
| 1. Lotsch 2021 | Identifying clinically relevant subgroups of patients with respect to olfactory loss during rhinitis and its recovery, with additional relevance to other sensory systems. | 117 | Supervised machine learning | ML algorithm assigns patients to subgroups based on olfactory function with accuracy of 64-65% | 3 |
| 1. Jabez Christopher 2015 | CDSS to assist junior clinicians in the diagnosis of AR | 872 | Association rule-based classification approach | CDSS diagnosed AR with accuracy of 88.31% | 4 |
| 1. Huang 2021 | Data mining methods used to predict childhood atopic dermatitis and allergic rhinitis using longitudinal birth cohort data | 1439 | ML: LR, RF, KNN, XGB, NB and NN | RF was the most accurate at diagnosing AD and AR with AUCs of 0.83 and 0.84 respectively | 4 |
| 1. Caimmi 2018 | Stratify patients suffering from seasonal AR and to propose cut-offs to identify severe forms of the disease | 28109 | KMC, AHC | KMC and AHC achieved overall AR misclassification rates of 4.30% and 22.39% respectively | 4 |

*AD: Atopic dermatitis; AHC: Agglomerative Hierarchical Clustering; AI: Artificial intelligence; AR: Allergic rhinitis; CDSS: clinical decision support system; CRS: Chronic Rhinosinusitis; KMC: k-Means Clustering; KNN: k-nearest neighbour; LOE: Level of evidence; LR: Logistic regression; ML: Machine Leaning; NB: Naïve Bayes; NN: Neural network; OD: Olfactory dysfunction; RF: Random Forest; SS: Sample Size; XGB: eXtreme Gradient Boost

**Appendix A.** Detailed Search Strategy for the State-of-the-Art Review

1. exp Artificial Intelligence/

2. exp Machine Learning/

3. exp neural networks, computer/

4. neural network*.mp.

5. Pattern Recognition, Automated/

6. Computer vision*.mp.

7. Robotic Surgical Procedures/

8. Robotic surg*.mp.

9. telesurgery.mp.

10. tele-surgery.mp.

11. Robotics/

12. Artificial intelligence.mp.

13. machine learning.mp.

14. deep learning.mp.

15. pattern recognition.mp.

16. AI.tw.

17. Robotic*.mp.

18. Decision Making, Computer-Assisted/

19. data science.mp.

20. 1 or 2 or 3 or 4 or 5 or 6 or 7 or 8 or 9 or 10 or 11 or 12 or 13 or 14 or 15 or 16 or 17 or 18 or 19

21. exp Otolaryngology/

22. Otolaryngology.mp.

23. Rhinology.mp.

24. exp Nose Diseases/

25. nose disease*.mp.

26. Paranasal sinus*.mp.

27. sinusitis/

28. rhinitis/

29. sinusitis.mp.

30. rhinitis.mp.

31. CRS.mp.

32. Chronic rhinosinusitis.mp.

33. 21 or 22 or 23 or 24 or 25 or 26 or 27 or 28 or 29 or 30 or 31 or 32

34. 20 and 33

**Supplement References**

1. Bur AM, Shew M, New J. Artificial Intelligence for the Otolaryngologist: A State of the Art Review. *Otolaryngol--Head Neck Surg Off J Am Acad Otolaryngol-Head Neck Surg*. 2019;160(4):603-611. doi:10.1177/0194599819827507

2. You E, Lin V, Mijovic T, Eskander A, Crowson MG. Artificial Intelligence Applications in Otology: A State of the Art Review. *Otolaryngol--Head Neck Surg Off J Am Acad Otolaryngol-Head Neck Surg*. 2020;163(6):1123-1133. doi:10.1177/0194599820931804

3. Crowson MG, Ranisau J, Eskander A, et al. A contemporary review of machine learning in otolaryngology-head and neck surgery. *The Laryngoscope*. 2020;130(1):45-51. doi:10.1002/lary.27850

4. Davenport T, Kalakota R. The potential for artificial intelligence in healthcare. *Future Healthc J*. 2019;6(2):94-98. doi:10.7861/futurehosp.6-2-94

5. Deo RC. Machine Learning in Medicine. *Circulation*. 2015;132(20):1920-1930. doi:10.1161/CIRCULATIONAHA.115.001593

6. Sekhar LN, Juric-Sekhar G, Qazi Z, et al. The Future of Skull Base Surgery: A View Through Tinted Glasses. *World Neurosurg*. 2020;142:29-42. doi:10.1016/j.wneu.2020.06.172

7. Deep Learning vs. Machine Learning — What’s the Difference? Flatiron School. Published February 8, 2021. Accessed May 16, 2022. https://flatironschool.com/blog/deep-learning-vs-machine-learning/

8. Etienne H, Hamdi S, Le Roux M, et al. Artificial intelligence in thoracic surgery: past, present, perspective and limits. *Eur Respir Rev Off J Eur Respir Soc*. 2020;29(157):200010. doi:10.1183/16000617.0010-2020

9. Chowdhury NI, Li P, Chandra RK, Turner JH. Baseline mucus cytokines predict 22-item Sino-Nasal Outcome Test results after endoscopic sinus surgery. *Int Forum Allergy Rhinol*. 2020;10(1):15-22. doi:10.1002/alr.22449

10. Thaler ER, Hanson CW. Use of an Electronic Nose to Diagnose Bacterial Sinusitis. *Am J Rhinol*. 2006;20(2):170-172. doi:10.1177/194589240602000209
